# Insecticidal Activity and Mechanism of 7-Methylcoumarin Against *Diaphorina citri* Reveal the Role of Chemosensory Genes in Defense Response

**DOI:** 10.3390/insects17101045

**Published:** 2026-10-12

**Authors:** Ao Fang, Xue-Yan Wang, Zheng-Hui Zou, Tong Yang, Wei Chen, Hai-Zhong Yu, Bing-Qi Liu, Ying-Xue Liu, Zhan-Jun Lu, Xiao-Qiang Liu

**Affiliations:** 1 National Navel Orange Engineering Research Center, College of Life Sciences, Gannan Normal University, Ganzhou 341000, China18363973378@163.com (B.-Q.L.); 2 Jiangxi Provincial Key Laboratory of Pest and Disease Control of Featured Horticultural Plants, Gannan Normal University, Ganzhou 341000, China

**Keywords:** *Diaphorina citri*, 7-methylcoumarin, transcriptome analysis, chemosensory genes, RNAi

## Abstract

The Asian citrus psyllid (*Diaphorina citri*) is a major vector of Huanglongbing (HLB), a devastating citrus disease worldwide. In addition to vectoring HLB, *D. citri* is a significant economic pest in its own right, causing direct damage to citrus by feeding on tender shoots, leading to leaf distortion, shoot dieback, and sooty mold infestation. Chemical control remains the primary strategy for managing *D. citri* populations, but heavy reliance on insecticides has led to insecticide resistance. Coumarin and its derivatives, as a natural product, are widely recognized for their insecticidal activities against various agricultural pests. In this study, the insecticidal activity of 11 coumarin compounds against *D. citri* nymphs was evaluated by bioassay, among which 7-methylcoumarin exhibited the most substantial efficacy. Moreover, the insecticidal mechanism of 7-methylcoumarin was assessed by a comparative transcriptome analysis, and 294 differentially expressed genes (DEGs) were identified, including 165 up-regulated and 129 down-regulated genes. GO enrichment analysis showed that DEGs were mainly involved in extracellular region/space, (chitin-based) cuticle development, and structural constituent of (chitin-based) cuticle terms, especially down-regulated genes related to cuticle proteins, chemosensory proteins and odorant-binding proteins, which were further verified by qPCR. Among chemosensory genes, only *DcCSP2* was significantly up-regulated, indicating its potential involvement in the defense response of *D. citri*. Suppression of *DcCSP2* via RNAi significantly increased susceptibility to 7-methylcoumarin in *D. citri* nymphs. Molecular docking revealed that 7-methylcoumarin binds within the internal hydrophobic cavity of DcCSP2. This finding provided a potential molecular mechanism underlying the action of coumarin compounds against pest insects and may contribute to facilitating the development of new strategies for managing insecticide resistance.

## Simple Summary

The Asian citrus psyllid (*Diaphorina citri*) is the main insect that spreads Huanglongbing, one of the most destructive diseases of citrus worldwide. Managing this pest relies heavily on chemical insecticides, but the psyllid has developed resistance to many of them. In this study, we tested 11 natural coumarin compounds against psyllid nymphs and found that 7-methylcoumarin was the most effective. Using comparative transcriptome analysis, we found that this compound suppresses genes related to the insect’s outer protective layer and its chemical-sensing proteins. We further showed that reducing the expression of *DcCSP2* made the nymphs much more susceptible to 7-methylcoumarin. These findings reveal how coumarins act against this pest and point to more environmentally friendly strategies for managing insecticide resistance in citrus orchards.

## 1. Introduction

The Asian citrus psyllid (*Diaphorina citri*) is a significant pest in the global citrus industry. It feeds on the phloem sap extensively and transmits the phloem bacterium (*Candidatus* Liberibacter asiaticus, *C*Las) through its saliva to citrus plants, ultimately causing the occurrence of Huanglongbing (HLB), also known as citrus greening disease [1]. HLB has caused irreversible losses to global citrus production, which presents characteristics such as poor fruit quality, low yield and short fruit lifespan [2]. Although outstanding progress has been made in HLB research [3], there is currently no effective method to completely cure this disease in the fields. Therefore, suppression of *D. citri* population via heavy application of broad-spectrum insecticides is one of the most principal strategies for HLB control [4]. However, the population of *D. citri* in different countries and regions has shown reduced susceptibility to many insecticides, including neonicotinoids, organophosphates, and pyrethroids, suggesting that this pest has developed resistance to different insecticides [5,6,7]. For example, *D. citri* field populations in Florida have developed high-level resistance to neonicotinoid insecticides such as imidacloprid (35.0-fold) and thiamethoxam (13.0-fold) due to the long-term use of these insecticides alone [8]. Field populations of *D. citri* from Ganzhou City, Jiangxi Province, China, showed high resistance to the insecticides chlorpyrifos, imidacloprid, dinotefuran, and bifenthrin, with resistance ratios of 507, 218, 139, and 95, respectively [7]. Consequently, the discovery of eco-friendly insecticides with new structures is important for *D. citri* resistance management.

Coumarin, a secondary metabolite initially isolated from the tonka bean (*Dipteryx odorata*), is widely distributed in various plants. It belongs to an important class of naturally occurring and synthetic oxygen-containing heterocycles, characterized by a fused benzene and α-pyrone ring system [9]. This distinctive benzopyran scaffold enables coumarin derivatives to readily interact with various enzymes and receptors in biological systems via non-covalent interactions, highlighting their promising therapeutic potential [10]. As a natural product, coumarins are safe, low-toxic, environmentally friendly, and do not easily develop resistance, and have high selectivity, and these unique characteristics have attracted great attention from researchers in the field of drug development [11]. Coumarin and its derivatives show a broad range of pharmacological effects, such as anti-inflammatory [12], anticancer [13], antiviral [14], antibacterial and antifungal activities [15,16]. Thus, their excellent properties significantly contribute to the application of novel medications.

Natural coumarins, including osthole, xanthotoxin, scopoletin, isoimperatorin and psoralen, have been found and isolated from plants. Among them, osthole derived from *Cnidium monnieri* has been extensively utilized in agriculture for its dual role as an insecticide and bacteriostatic compound [17]. Moreover, coumarin derivatives have attracted considerable interest as eco-friendly alternatives to traditional chemical insecticides due to their exceptional insecticidal, acaricidal, and nematicidal activities [11]. For example, coumarin derived from *Ageratum conyzoides* has displayed promising insecticidal efficacy against multiple pests like *Periplaneta americana*, *Diaphania hyalinata* and *Rhyzopertha dominica* [18]. In addition, the growth and survival of *Hyphantria cunea* larvae are markedly suppressed by coumarins [19]. Exposure to osthole resulted in observable cuticular damage and obvious growth-inhibitory activity in the mite *Tetranychus cinnabarinus* [20]. 6-Methylcoumarin exhibited significant insecticidal efficacy against *Aphis gossypii* (LC_50_ = 65.30 mg/L) through acetylcholinesterase (AChE) inhibition, while showing low toxicity to bees and mammals [21]. Recent research found that coumarin derivatives display potent antiviral activity against tobacco mosaic virus (TMV), while also exhibiting significant insecticidal effects against *Mythimna separate* [22]. Therefore, coumarin and its derivatives demonstrate significant potential for development as novel botanical insecticides in integrated pest management.

Due to the long-term use of traditional insecticides, the resistance of pests has increased, coumarin compounds have attracted much attention as environmentally friendly alternatives to traditional chemical insecticides. However, the biological activities of coumarin compounds against *D. citri* remain unclear. To evaluate the structure–activity relationships of coumarins against *D. citri* nymphs, a panel of 11 coumarin compounds with different substituent types and positions (e.g., hydroxyl and methyl groups at C4, C6, and C7) was selected, and their insecticidal activities were evaluated using the leaf-dipping bioassay method. Among the 11 tested coumarins, 7-methylcoumarin exhibited the highest insecticidal activity (73.23% mortality at 48 h) and was therefore selected for the subsequent mechanistic studies. The sub-lethal concentration of 7-methylcoumarin was further calculated, and then the differentially expressed genes were identified and analyzed by transcriptome sequencing. Furthermore, the function of chemosensory protein 2 was verified by RNA inference and bioassay. The findings of this study provide critical insights for developing effective field resistance management strategies against *D. citri* populations.

## 2. Materials and Methods

### 2.1. Psyllid Rearing

Asian citrus psyllid populations were reared on orange jasmine (*Murraya paniculata*) in mesh cages (60 × 60 × 90 cm) under controlled conditions: maintained at a temperature of 27 ± 1 °C, a relative humidity of 70 ± 5%, with a 14:10 h (light: dark) photoperiod in a climate-controlled growth chamber.

### 2.2. Chemicals and Reagents

Coumarin (CAS number: 91-64-5), scoparone (CAS number: 120-08-1), 7-hydroxycoumarin (CAS number: 93-35-6), daphnetin (CAS number: 486-35-1), 7-methylcoumarin (CAS number: 2445-83-2), xanthotoxin (CAS number: 298-81-7), 4-hydroxycoumarin (CAS number: 1076-38-6) and 6-hydroxycoumarin (CAS number: 6093-68-1) were bought from Naturell (Sichuan, China). 7,8-dihydroxy-4-phenylcoumarin (CAS number: 568-90-7), 7-amino-4-methylcoumarin (CAS number: 26093-31-2), and 7-mercapto-4-methylcoumarin (CAS number: 137215-27-1) were purchased from Merck (Darmstadt, Germany). Acetone and Tween-20 were purchased from Shanghai Richjoint Chemical Reagents Co., Ltd. (Shanghai, China). All chemicals and reagents were of analytical grade and had a purity over 90%.

### 2.3. Bioassays

To assess the insecticidal efficacy of eleven coumarin compounds against *D. citri* nymphs, a leaf-dipping bioassay was employed based on the IRAC Test Method No. 032, with minor modifications. Briefly, the coumarin compounds were dissolved in acetone and subsequently diluted in deionized water containing 0.1% Tween-20 to achieve a final concentration of 1 mg/mL. Then, 25–30 fifth-instar nymphs were placed on an orange jasmine (*Murraya paniculata*) leaf and immediately immersed in the coumarin solution for 30 s, followed by air-drying for 30 min at room temperature. Finally, the treated nymphs were transferred into an acrylic cage (12 × 12 × 32 cm) containing fresh orange jasmine leaves. The mortality of *D. citri* nymphs was recorded at 24 and 48 h post-treatment. Psyllids in the negative control group were treated with an equivalent volume of acetone solution (without the active compounds). Each coumarin compound was tested with four independent biological replicates.

### 2.4. RNA Extraction and Sequencing

Approximately 160 fifth-instar nymphs allocated into four independent replicates were treated with 7-methylcoumarin (0.4 mg/mL) as previously described. After 48 h treatment, ten surviving individuals for each group were collected for transcriptome analysis. Total RNA was isolated using the TRIzol Reagent (Invitrogen, Carlsbad, CA, USA), after which the concentration, integrity and quality were determined using a NanoDrop spectrophotometer (Thermo Scientific, Waltham, MA, USA) and agarose gel electrophoresis. A total of 3 μg RNA was used as input material for the RNA sample preparations. Sequencing libraries were constructed following the subsequent procedures. Initially, mRNA was purified from total RNA through the employment of poly-T oligo-attached magnetic beads. First-strand and second-strand cDNA were synthesized respectively, and to isolate cDNA fragments with a preferred length of 400–500 bp, the library fragments were purified by means of the AMPure XP system (Beckman Coulter, Beverly, CA, USA). DNA fragments with ligated adaptor molecules at both ends were selectively amplified. The reaction products were subsequently purified through the AMPure XP system and quantified using the Agilent high-sensitivity DNA assay on a Bioanalyzer 2100 system (Agilent, Santa Clara, CA, USA). Finally, the sequencing library was subjected to sequencing on the NovaSeq Xplus platform (Illumina, San Diego, CA, USA).

### 2.5. DEGs Analysis

After removing the sequences with adapters, N-bases and low-quality reads from the raw data, high-quality clean reads were obtained using Trimmomatic (Version 0.39). The expression level of genes was calculated by Salmon software and standardized by the Fragments Per Kilobase of exon per Million mapped fragments (FPKM) method. DEGs were analyzed by the DESeq2 R package (Version 1.40.2), and the screening condition was set as Q-value < 0.05. Meanwhile, gene ontology (GO) enrichment analysis was used to identify the function of DEGs using the topGO R package. Additionally, the Kyoto Encyclopedia of Genes and Genomes (KEGG) was performed to identify important pathways.

### 2.6. Quantitative Real-Time PCR (qPCR)

Ten *D. citri* nymphs treated with 7-methylcoumarin and acetone were collected. Total RNA was extracted using Trizol reagent, and the cDNA was prepared using Hifair AdvanceFast one-step RT-gDNA Digestion SuperMix (Yesen, Shanghai, China) according to the manufacturer’s instructions. The specific primers of DEGs were designed using Primer 3.0 (http://primer3.ut.ee/, accessed on 8 October 2026), which is shown in Table S1. The reaction volume is 10 μL, including 5 μL NovoStar SYBR qPCR SuperMix (Novoprotein Scientific, Shanghai, China), 0.5 μL cDNA (200 ng/μL), 0.5 μL of each primer (10 μM) and 2.5 μL ddH_2_O. qPCR was performed on the LightCycle 480 PCR Detection System (Roche, Basel, Switzerland). The procedure is as follows: 95 °C for 2 min; 40 cycles of 95 °C for 15 s and 60 °C for 30 s; concluding with a melting curve analysis (60–95 °C). Each experiment was performed with three biological replicates. The relative expression levels were normalized to the reference gene *DcGAPDH* (XM_008481620) and calculated using the 2^−ΔΔCt^ method [23,24].

### 2.7. Knockdown of DcCSP2 by RNAi

Based on the RNA-seq and qPCR results, the chemosensory protein 2 (*CSP2*) gene was further selected to investigate its role in regulating cuticle structure. The specific primers with T7 promoter sequence (Table S1) were designed and double-stranded RNA (dsRNA) of CSP2 was synthesized using a T7 RioMAX™ Express RNAi System (Promega, Madison, WI, USA) in vitro. RNA interference was performed in fifth-instar nymphs by microinjection in accordance with our previous research [25]. In brief, *dsCSP2* and dsGFP (control) were each prepared at 1500 ng/μL. For each treatment, 40 nL of dsRNA (60 ng) was injected into the ventral abdomen of fifth-instar *D. citri* nymphs. This concentration was consistent between the RNAi efficiency assay and the mortality test, and the same concentration was used for all genes, including the dsGFP control. A total of 200 nymphs divided into five biological replicates were injected for each treatment. To evaluate RNAi efficacy, eight nymphs were collected at 24, 48 and 72 h post-injection. The procedures for total RNA extraction, cDNA synthesis and RT-qPCR assay were carried out as previously described. For the mortality assay, about 300 nymphs were allocated to six biological replicates per treatment and then were subjected to injection. The mortality of *D. citri* was assessed at 12 h intervals throughout a period of 3 consecutive days.

### 2.8. Molecular Docking

Protein–ligand complex prediction was carried out using Boltz-2. The full-length amino acid sequences of DcCSP2 in FASTA format were used as input, and the three-dimensional structure of 7-methylcoumarin was defined by its SMILES string. Calculations were run with the following parameters: recycling_steps = 6, sampling_steps = 200, diffusion_samples = 5, and step_scale = 1.0; all other parameters were left at their default values. Predicted complex conformations were ranked by confidence scores, and the top-scoring model was selected for downstream interaction analysis. Structural visualization and interaction analysis were performed using PyMOL (Version 3.1.0) (Schrödinger, LLC, New York, NY, USA).

### 2.9. Statistical Analysis

All statistical analysis was performed using IBM SPSS Statistics 20.0 software. Data from all experiments were expressed as mean ± standard errors (SE). The comparison of multiple groups was performed using one-way analysis of variance (ANOVA) with post hoc Tukey’s honestly significant difference (HSD) test (*p* < 0.05). The probit analysis was used to calculate the median lethal concentration (LC50) of 7-methylcoumarin against *D. citri* nymphs. Moreover, the significant difference between two independent groups was evaluated using Student’s *t*-test (*p* < 0.05).

## 3. Results

### 3.1. Insecticidal Activity of Coumarin Compounds Against D. citri

**Figure 1:**
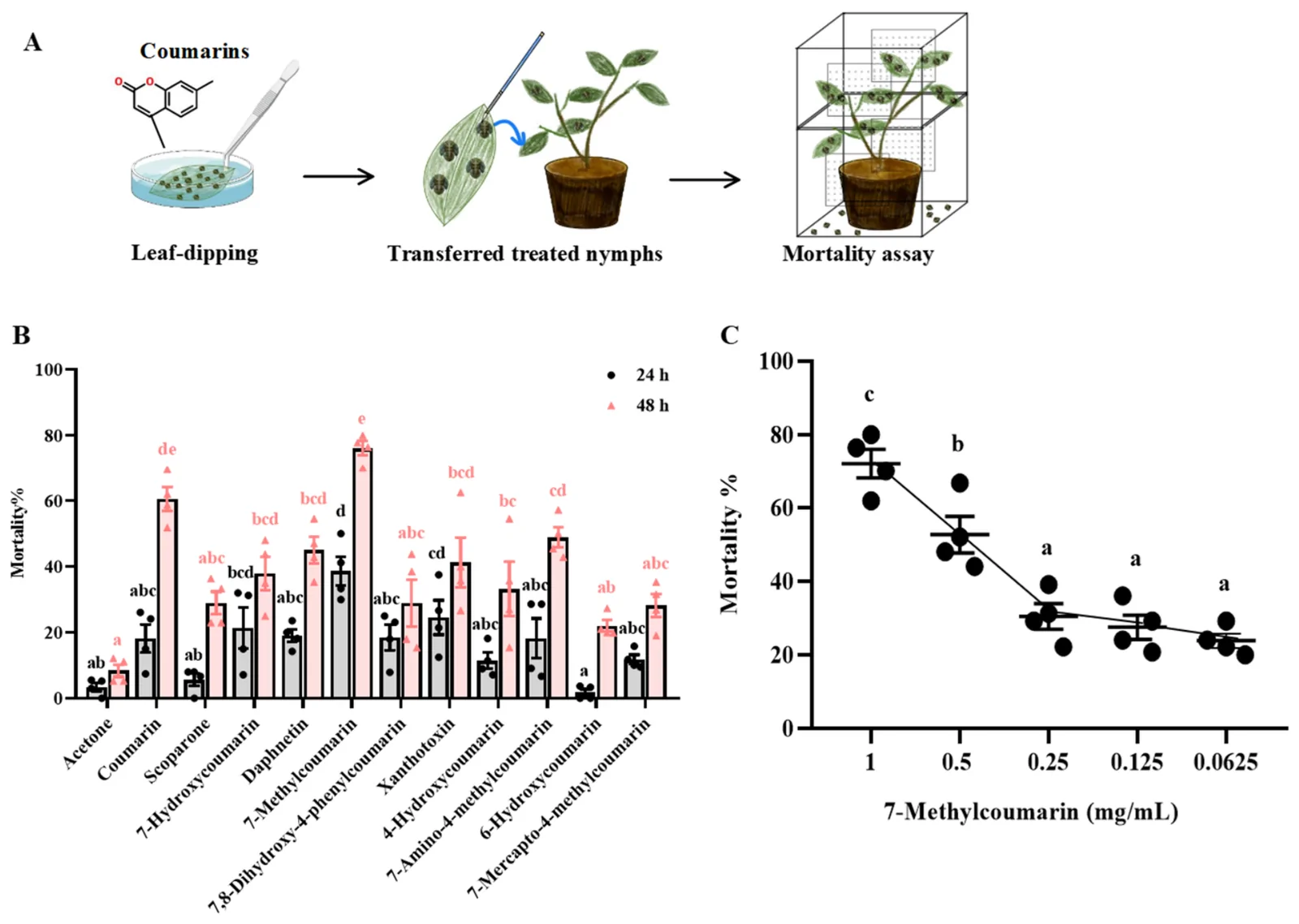
Determination of the insecticidal activity of coumarin compounds against *D. citri* nymphs. (**A**) Flow chart of coumarin treatment against nymphs by the leaf-dipping method. (**B**) Insecticidal activity of 11 coumarin compounds against *D. citri* nymphs at 24 h (gray bars) and 48 h (pink bars). The tested concentration of each coumarin compound was 1 mg/mL. Mortality was corrected using Abbott’s formula based on the corresponding acetone control. (**C**) The susceptibility assay of *D. citri* nymphs to different concentrations of 7-methylcoumarin. The vertical bars indicate the standard error (SE) of the mean. Different letters above the bars represent significant differences among the coumarin compound treatments as determined by one-way analysis of variance (HSD, *p* < 0.05, *n* = 4).

To investigate the bioactivities of coumarin compounds on the mortality of *D. citri*, the insecticidal activities of eleven coumarins against *D. citri* nymphs were tested. The experimental flow chart for the insecticidal effect assay of coumarins was shown in Figure 1A. The results showed that different coumarin compounds exhibited different insecticidal activities (Figure 1B). At 24 h after treatment with coumarin compounds, the mortality rates of *D. citri* nymphs were relatively low, with most below 30%, except for 7-methylcoumarin (31.86%). However, after 48 h of treatment, the 7-methylcoumarin treatment demonstrated superior insecticidal efficacy, achieving 73.23% mortality, which was the highest among all test compounds. Additionally, susceptibility of *D. citri* nymphs to 7-methylcoumarin diminished progressively with decreasing concentration (Figure 1C). The LC_50_ of 7-methylcoumarin against *D. citri* nymphs was 0.4 mg/mL at 48 h (Table S2), suggesting that 7-methylcoumarin has potential as a botanical insecticide against *D. citri*.

### 3.2. DEGs Analysis

Eight samples (four control groups, namely Ace1–4, and four treatment groups, namely m7Cou1–4) were sequenced by the NovaSeq Xplus platform (Illumina). After removing the low-quality reads, a total of 38.63–50.40 million clean reads for the control groups and 40.70–54.72 million clean reads for the treatment groups were obtained, respectively (Table S3). In addition, the values of Q20 (Phred score > 20) and Q30 (Phred score > 30) exceeded 99% and 96%, respectively. Overall, these results suggested that the sequencing quality was reliable. To investigate the potential functions of unigenes in *D. citri*, FPKM values of unigenes were compared between control and treatment groups. In summary, 294 differentially expressed genes (DEGs) exhibited significant variations, including 165 up-regulated and 129 down-regulated genes, as shown in Figure S1A and Table S4. The hierarchical clustering of all DEGs was performed to analyze their expression patterns (Figure S1B).

**Figure 2:**
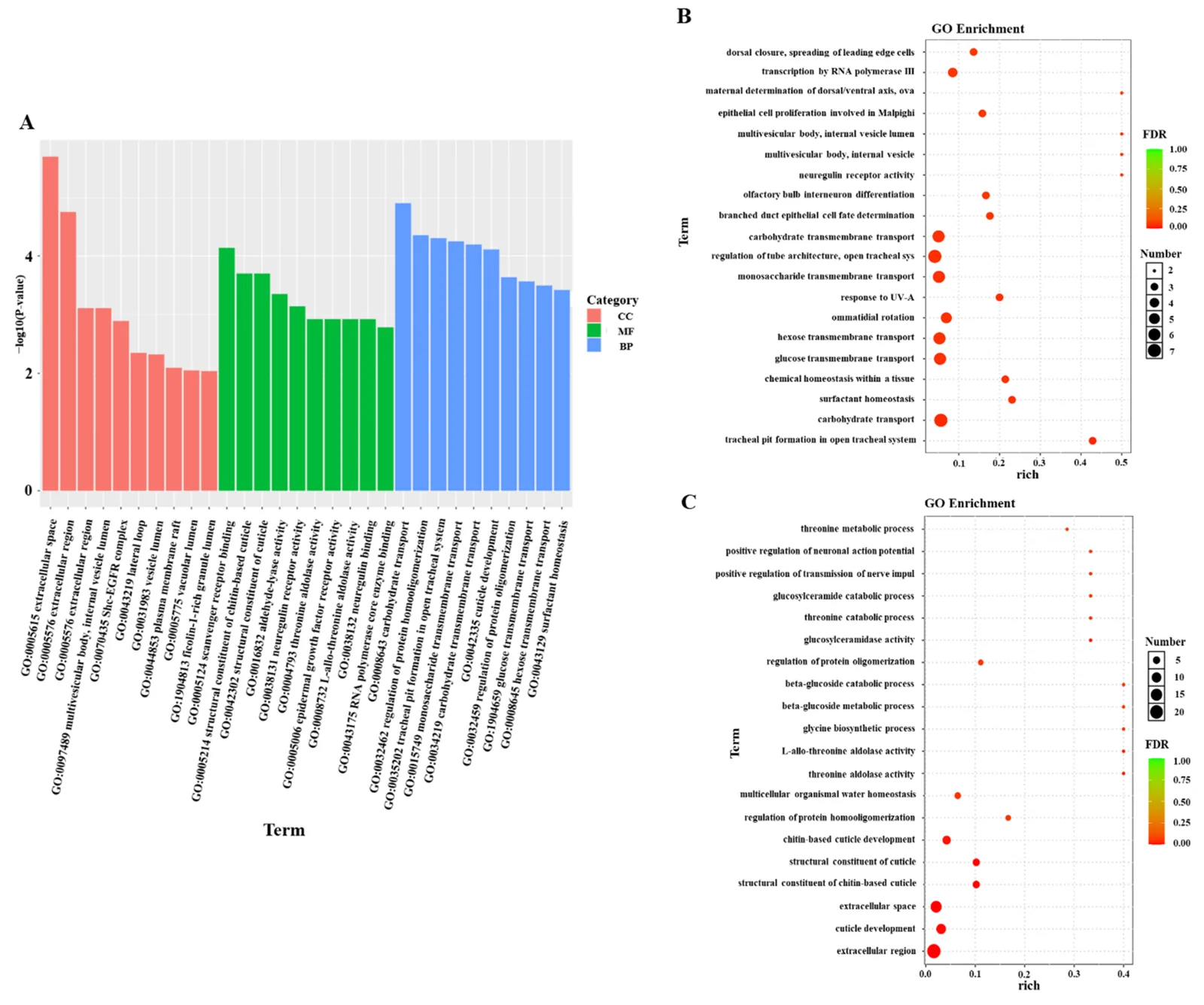
GO analysis of DEGs. (**A**) GO function classification. The results were functionally grouped into three categories: CC (cellular component), MF (molecular function) and BP (biological process). For each category, the top 10 GO terms were used for functional analysis. (**B**) GO enrichment in up-regulated genes and (**C**) GO enrichment in down-regulated genes. The top 20 GO terms were used for enrichment analysis. The color and size of bubbles refer to the False Discovery Rate (FDR) values and gene numbers.

The results of Gene Ontology (GO) enrichment analysis for the DEGs were classified into three categories: cellular component (CC), molecular function (MF), and biological process (BP). For each category, the 10 most significantly enriched GO terms (Top 10) were further analyzed (Figure 2A). In the CC category, extracellular space and extracellular region had the most abundant DEGs; in the MF category, scavenger receptor binding, structural constituent of chitin-based cuticle and structural constituent of cuticle possessed the most abundant DEGs; in the BP category, carbohydrate transport was the most abundant. Based on the GO enrichment results, the degree of enrichment was evaluated by the Rich factor, FDR value and the number of genes enriched in this GO term. Among them, up-regulated DEGs related to regulation of tube architecture, open tracheal system, carbohydrate transport, carbohydrate transmembrane transport, monosaccharide transmembrane transport, glucose transmembrane transport and hexose transmembrane transport were mainly enriched (Figure 2B), while down-regulated DEGs were mainly enriched in extracellular region, extracellular space, cuticle development, structural constituent of chitin-based cuticle, structural constituent of cuticle, and chitin-based cuticle development (Figure 2C). According to the Kyoto Encyclopedia of Genes and Genomes Pathway database (KEGG) enrichment analysis, different metabolic pathways are divided into five categories, including Cellular Processes, Environmental Information Processing, Genetic Information Processing, Metabolism, and Organismal Systems (Figure S2).

Subsequently, to reveal the potential molecular mechanism of toxicity activity of 7-methylcoumarin against *D. citri* nymphs, the DEGs related to chemosensory, cuticular and metabolic detoxification systems, such as odorant-binding proteins (OBPs, Dcitr08g02940), chemosensory proteins (CSPs, Dcitr02g16710, Dcitr13g06590, Dcitr13g06620, Dcitr02g18920), cuticle proteins (Dcitr08g07270, Dcitr08g07890, Dcitr08g04430, Dcitr03g21110) and cytochrome P450 enzymes (CYPs, Dcitr01g05740, Dcitr01g05750) were searched and compared (Table S4). Especially, most DEGs involved in those selected systems were down-regulated with log2-fold change values from −1.20 to −2.00; only one CSP gene (Dcitr02g18920, CSP2) was up-regulated with a log2-fold change value of 2.81.

### 3.3. qPCR Validation

**Figure 3:**
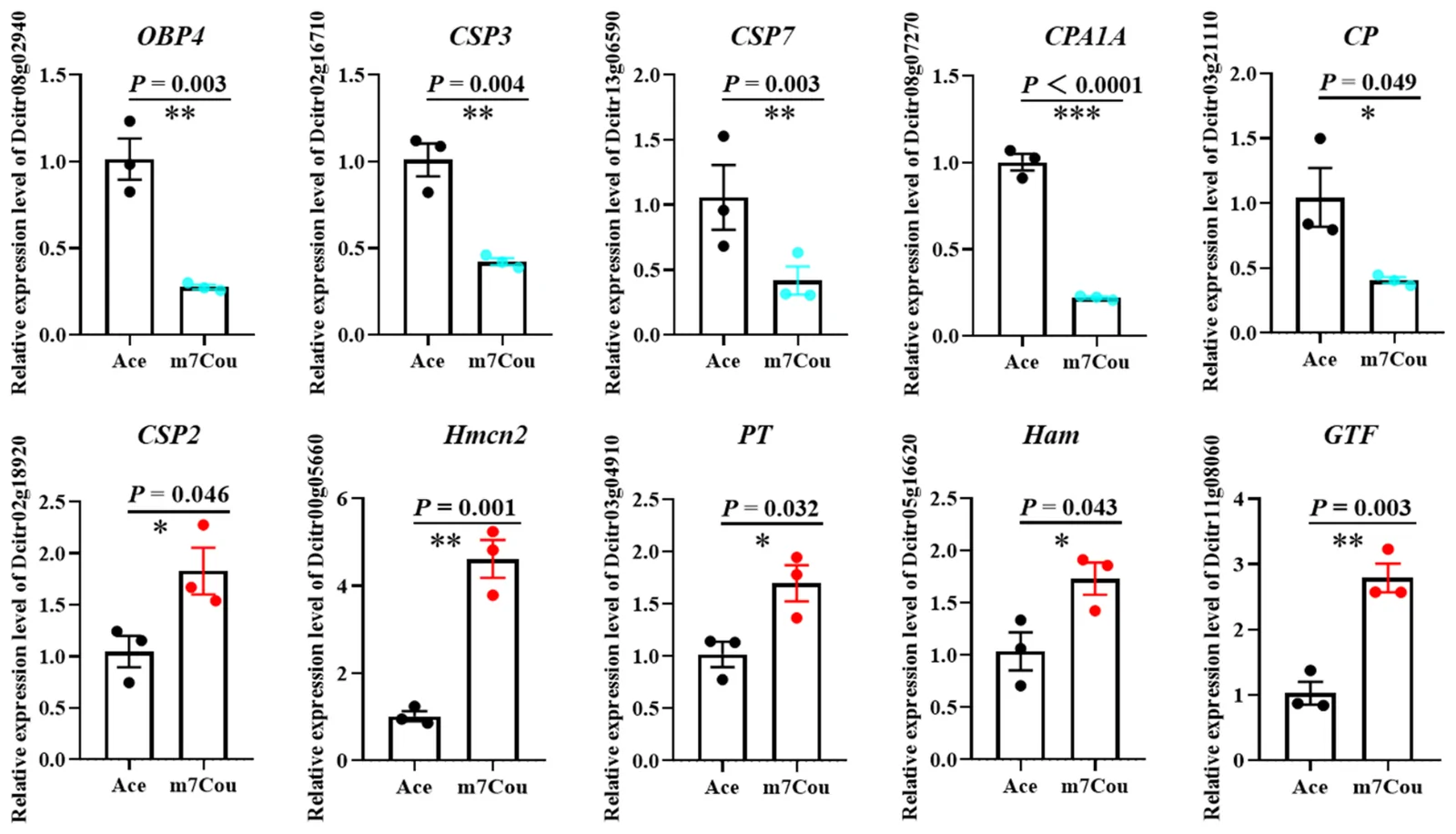
Validation of RNA-seq data by qPCR for 10 selected DEGs (five down-regulated and five up-regulated genes) covering chemosensory, cuticle-related, and detoxification-related functional categories. Relative expression levels were normalized to the reference gene *DcGAPDH* using the 2^−ΔΔCt^ method, with the acetone-treated group as the calibrator. Data are represented as the mean ± SE from three independent replicates. Significant differences are indicated by * *p* < 0.05, ** *p* < 0.01 and *** *p* < 0.001 (Student’s *t*-test).

To validate the RNA-seq data, 10 differentially expressed genes (DEGs), including five downregulated and five upregulated genes covering chemosensory (OBP/CSP), cuticle-related, and detoxification-related functional categories, were selected and validated by RT-qPCR. The information on selected DEGs is shown in Table 1. Expectedly, the relative transcript levels were highly consistent with the results of RNA-seq (Figure 3). For example, 5 genes exhibited significant downregulation upon 7-methylcoumarin treatment in both qPCR and RNA-seq analysis. Five selected genes were significantly increased after 7-methylcoumarin treatment in both qPCR and transcriptome analysis. Overall, the qPCR results validated the credibility and reliability of the RNA-seq data.

### 3.4. Silencing DcCSP2 Increased the Susceptibility of D. citri Response to 7-Methylcoumarin

**Figure 4:**
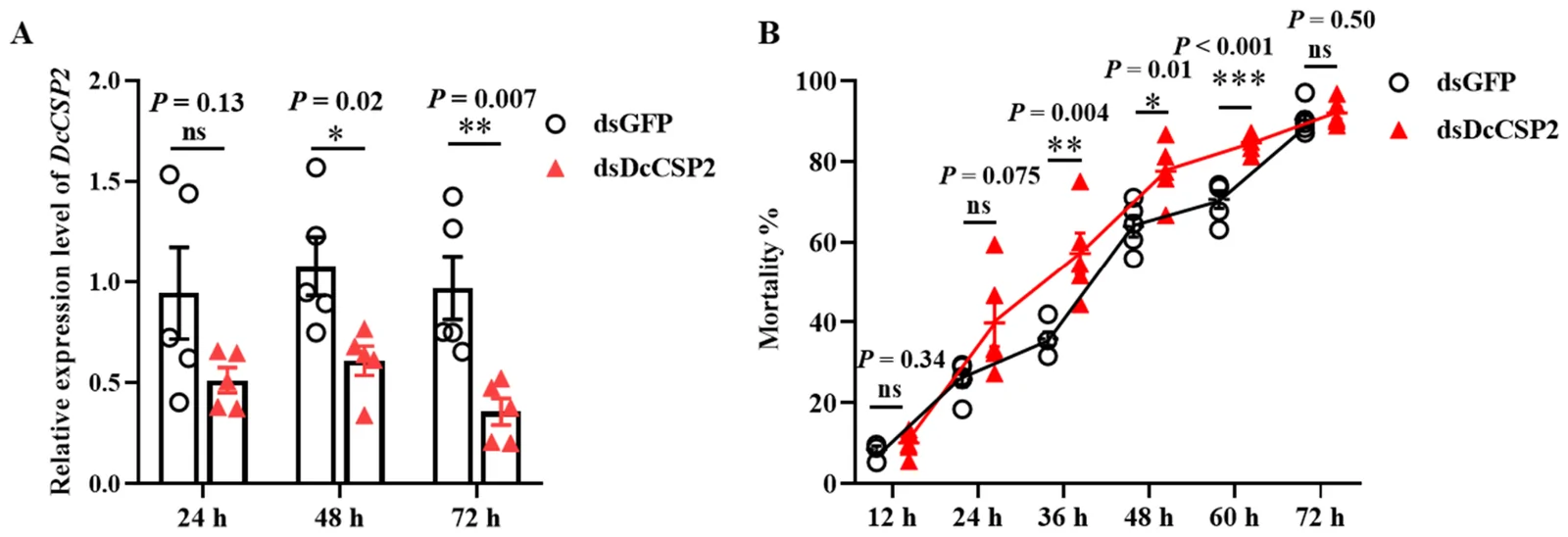
Effect of *DcCSP2* silencing by RNAi on *D. citri* nymphs. (**A**) The expression changes in *DcCSP2* were examined at 24, 48 and 72 h after injection with *dsDcCSP2* via RT-qPCR. The dsRNA concentration was 1500 ng/μL. The 2^−ΔΔCt^ method was used to calculate relative expression levels, with DcGAPDH as the reference gene. (**B**) Suppression of *DcCSP2* increased the susceptibility of *D. citri* nymphs to 7-methylcoumarin. The dsRNA concentration was 1500 ng/μL. Data are shown as the mean ± SE (*n* = 5). Significant differences were assessed using Student’s *t*-test (* *p* < 0.05, ** *p* < 0.01, *** *p* < 0.001 and ns indicates no significant difference).

To investigate the role of *DcCSP2* in coumarin resistance, RNAi was performed by microinjection of dsRNA. As shown in Figure 4A, a markedly lower transcript level of *DcCSP2* was observed after injection of *dsDcCSP2* compared with the dsGFP control. At 24 h post-injection, the relative expression of *DcCSP2* decreased by 45.69% in the *dsDcCSP2* treatment, but this decrease was not significantly different from that of the control (*p* = 0.13). However, *DcCSP2* expression levels treated with *dsDcCSP2* were significantly inhibited, with reductions of 43.56% (*p* = 0.02) and 63.11% (*p* = 0.007) at 48 and 72 h, respectively. After *dsDcCSP2* injection, the mortality of *D. citri* nymphs upon exposure to 7-methylcoumarin was significantly elevated compared with the dsGFP control, with an increase of 21.20% (*p* = 0.004), 13.64% (*p* = 0.01) and 14.09% (*p* < 0.001) at 36 h, 48 h and 60 h, respectively (Figure 4B). Additionally, the mortality caused by *dsDcCSP2* injection was higher than that of the control at 12, 24 and 72 h, but the differences were not statistically significant.

### 3.5. Molecular Docking Reveals the Binding Mode of 7-Methylcoumarin to DcCSP2

**Figure 5:**
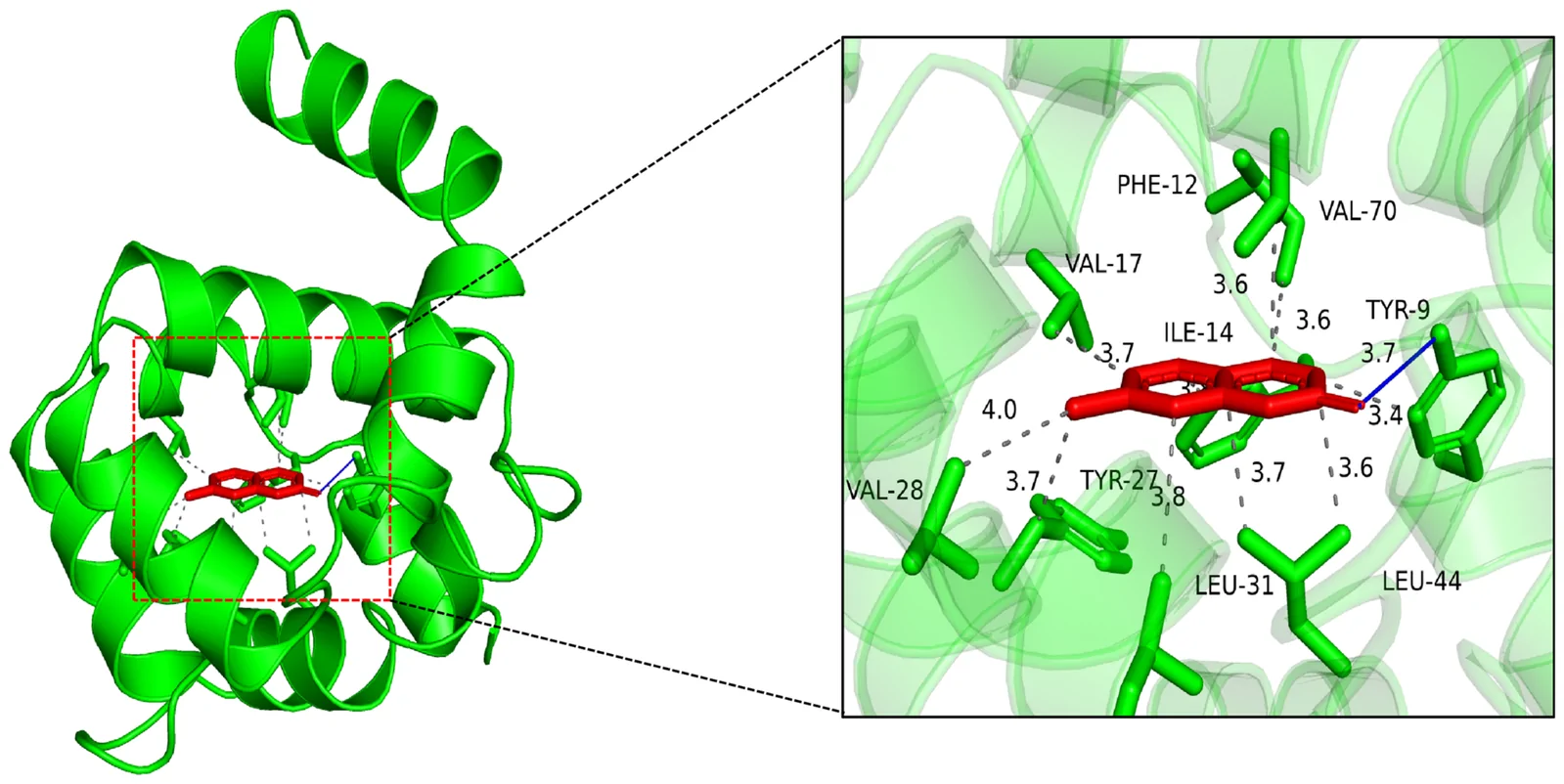
Molecular docking analysis of 7-methylcoumarin with DcCSP2. The binding pose of 7-methylcoumarin (red) accommodated in the hydrophobic pocket of DcCSP2 (green) was predicted by Boltz-2. Blue solid lines denote hydrogen-bond interactions, and gray dashed lines indicate hydrophobic contacts between the ligand and the surrounding amino-acid residues.

Molecular docking was performed to explore the binding modes of 7-methylcoumarin with the olfactory-related protein DcCSP2. As shown in Figure 5, 7-methylcoumarin occupies the internal hydrophobic cavity of DcCSP2. The oxygen atom of the 7-methylcoumarin lactone ring forms a hydrogen bond with the hydroxyl group of Tyr9 in DcCSP2. This polar interaction contributes to ligand-binding specificity. The 7-methylcoumarin core is surrounded by a cluster of hydrophobic residues, including Tyr9, Phe12, Ile14, Val17, Tyr27, Val28, Leu31, Leu44, and Val70, which together form extensive hydrophobic contacts. The reliability of the predicted complex model was further supported by the high confidence scores: the complex pLDDT, iPTM, pTM, and structure confidence were 0.87, 0.90, 0.89, and 0.88 for the DcCSP2–7-methylcoumarin complex, with a predicted docking energy (pDE) value of 0.48. Collectively, these results indicate that 7-methylcoumarin can stably bind within the hydrophobic cavity of DcCSP2, providing a structural basis for the recognition and sequestration of this compound by this olfactory-related protein.

## 4. Discussion

Currently, substantial quantities of synthetic chemical pesticides have been employed to control crop diseases and pests; unfortunately, the increasing prevalence of pesticide resistance and residue accumulation has emerged as a worldwide challenge to human health and ecological safety [26,27]. Thus, to address the problems posed by conventional pesticides, there is a critical need to develop novel, environmentally benign, and highly efficacious alternatives with low ecological risk. Plant natural products, which encompass plant derivatives, crude extracts, and bioactive compounds, have significant advantages such as wide availability, structural diversity, biodegradability and low pesticide resistance [22]. Among them, coumarins, as a promising group of naturally derived bioactive heterocyclic compounds, have unique pharmacological, biological, and physiological properties in many fields [11]. For example, osthole, a natural coumarin compound, is widely employed in agriculture for its insecticidal and acaricidal properties [28]. In the current study, we evaluated the insecticidal activity of 11 coumarin compounds against *D. citri* and found that 7-methylcoumarin showed the highest mortality (73.23% at 48 h) among the tested compounds, with an LC_50_ of 0.40 mg/mL (400 mg/L) at 48 h. Although this value is moderate compared with commercial chemical insecticides, 7-methylcoumarin exhibited considerable insecticidal activity among the tested natural coumarin compounds and can be regarded as a promising lead compound for the further development of botanical insecticides against *D. citri*. Recent studies have reported that coumarin compounds have impressive insecticidal activity against various pests, including *Myzus persicae* [29], *H. cunea* [19], red palm weevils *Rhynchophorus ferrugineus* [30], *Spodoptera litura* [31], and *Plutella xylostella* [32]. To our knowledge, this study provides the first report on the insecticidal activity of coumarin against *D. citri*. Modifications at the C4 and C7 positions of the coumarin scaffold are known to enhance bioactivity, making them promising sites for the development of novel active compounds [11]. Our results also showed that methylation modification of the C7 position of the coumarin exhibits distinctly higher insecticidal activity than other tested coumarin compounds. Similarly, the C7 position bearing a hydroxyl group showed high insecticidal activity against *M. separate* [22]. In addition, 6-methylcoumarin exhibited strong aphicidal activity, with an efficacy comparable to that of the commercial insecticide malathion [21]. These results suggested that coumarin and its derivatives show prospects as potent botanical insecticides for pest management.

GO enrichment analysis showed that DEGs were mainly connected with the extracellular region/space, (chitin-based) cuticle development, and structural constituents of the (chitin-based) cuticle in *D. citri* after 7-methylcoumarin treatment. Among them, DEGs related to cuticle development, such as cuticle protein (CP) genes, were significantly decreased after application of 7-methylcoumarin. Our findings suggest that 7-methylcoumarin might suppress the expression of cuticle-related genes and destroy the structural constituents of the cuticle to enhance susceptibility in *D. citri* nymphs. A similar result was observed in *T. cinnabarinus*, where scanning electron microscopy (SEM) revealed that the osthole derivative caused structural disruption of the mite cuticle [20]. However, Transcriptomic analysis identified numerous detoxification-related DEGs, including CYPs, GSTs and esterases, in *S. litura* larvae after coumarin treatment [31]. These findings suggested that coumarin compounds elicit distinct molecular responses across different insect species. In insects, certain structural components, including chitin and cuticular proteins, serve critical functions in protecting against pathogenic invasions and limiting the penetration of toxic substances [33]. Moreover, the cuticle serves as the primary and initial defensive barrier, preventing the penetration of external substances into insects. A large number of cuticle protein genes (up-regulated or down-regulated) were identified through comparative transcriptome analysis after validamycin exposure to *D. citri* nymphs, and RNA interference further revealed that silencing the expression of DcCP7 genes resulted in an increase in mortality and malformations [34]. Taken together, the evidence indicates that coumarin-derived compounds impair cuticular formation and structural integrity by downregulating cuticle protein genes, thereby compromising the cuticle’s barrier function and increasing pest susceptibility. However, whether 7-methylcoumarin directly disrupts the cuticular structure of *D. citri* nymph requires further investigation.

It is well established that chemosensory genes like OBPs and CSPs are essential for the recognition and transport of chemical agents, allowing insects to perceive external stimuli and mount appropriate behavioral and physiological responses to environmental changes [35,36]. Recently, OBPs and CSPs have received increasing attention for their potential roles in the development of insecticide resistance, largely due to their capacity to bind and sequester insecticidal compounds, thereby reducing their effective toxicity [37,38]. Moreover, CSP-mediated regulation has been proposed as a novel mechanism underlying insecticide resistance in insects [39]. In the present study, one OBP and two CSP genes were significantly down-regulated based on transcriptome analysis, indicating that their binding capacities to 7-methylcoumarin were weakened, which seemed to ultimately diminish the resistance of *D. citri* to this compound. Interestingly, under the stress of 7-methylcoumarin, only the expression of *DcCSP2* was significantly up-regulated among these chemosensory genes, implying that it may be specifically involved in the defense mechanisms of *D. citri*. Molecular docking revealed that 7-methylcoumarin binds within the internal hydrophobic cavity of DcCSP2, and Tyr9 participates in both hydrogen bonding and hydrophobic stacking, making it a key residue for ligand recognition and binding. However, further studies are needed to determine whether DcCSP2 binds to 7-methylcoumarin.

We further investigated the function of *DcCSP2* by RNAi; suppression of *DcCSP2* resulted in high mortality of *D. citri* nymphs upon exposure to 7-methylcoumarin. Similarly, transcriptome analysis in *Tribolium castaneum* revealed that the expression of many DEGs, including those encoding OBP and CSP genes, was significantly altered in response to fumigation with citronellyl formate, and RNAi targeting TcGOBP70 dramatically increased the mortality of the beetles to this chemical [40]. Knockdown of CSPs impaired the capacity of *Nilaparvata lugens* to metabolize imidacloprid and inhibited the activity of metabolic detoxification pathways, thereby reducing imidacloprid resistance [41]. In previous studies, silencing of *SAP2* (a *CSP* gene) was found to fully restore pyrethroid-induced mortality in *Anopheles gambiae*, whereas its overexpression conferred increased resistance by binding pyrethroid insecticides with high affinity [39]. Numerous previous studies have demonstrated that the expression of OBP and CSP genes can be induced by insecticides or secondary metabolites in various insect species, and knocking down their transcript levels resulted in increased susceptibility of insects to such chemical agents [25,37,42,43], underscoring their critical role in detoxification and adaptive response [24,41]. Consequently, CSPs or OBPs play a conserved and critical role in modulating insecticidal active substances resistance across species, offering potential molecular targets for the development of novel pest control strategies.

## 5. Conclusions

The insecticidal activity of 11 coumarin compounds against *D. citri* nymphs was evaluated, and 7-methylcoumarin exhibited the most substantial toxicity. Transcriptome analysis combined with qPCR validation revealed that 7-methylcoumarin significantly inhibited the expression of cuticle development-related genes and chemosensory genes. Knockdown of *DcCSP2* enhanced the susceptibility of *D. citri* nymphs to 7-methylcoumarin. These findings elucidate the multifaceted toxicity of coumarin compounds and provide a molecular basis for the development of novel botanical insecticides targeting psyllid pests.

## Figures and Tables

**Table 1:** Information on the 10 DEGs used in qPCR validation.

Gene ID	Gene Name	Ace	m7Cou	log_2_FoldChange	*p*-Value
Dcitr08g02940	Odorant binding protein 4	1828.30	461.04	−1.99	0.014
Dcitr02g16710	Chemosensory protein 3	4664.66	2017.03	−1.21	0.00025
Dcitr13g06590	Chemosensory protein 7	24.47	9.77	−1.3	0.015
Dcitr08g07270	Larval cuticle protein A1A	8181.10	2267.59	−1.85	0.015
Dcitr03g21110	Cuticle protein	1339.18	485.71	−1.46	0.019
Dcitr02g18920	Chemosensory protein 2	1.37	9.63	2.81	0.014
Dcitr00g05660	Hemicentin-2	14.39	33.63	1.23	0.013
Dcitr03g04910	Phosphotransferase	8.09	20.63	1.35	0.018
Dcitr05g16620	Hamartin	9.42	23.71	1.33	0.013
Dcitr11g08060	General transcription factor 3C	0.52	4.45	3.10	0.025

## Data Availability

The transcriptome sequencing data reported in this study will be deposited in the NCBI Sequence Read Archive (SRA) under accession number [PRJNA1529345]. Other data supporting the findings of this study are available from the corresponding author upon reasonable request.

## Supplementary Materials

The following supporting information can be downloaded at https://www.mdpi.com/article/10.3390/insects17101045/s1: Table S1: Primers used in this study; Table S2: Median lethal concentration (LC_50_) of 7-methylcoumarin against *D. citri* nymphs; Table S3: Summary of transcriptome sequencing data; Table S4: Differentially expressed genes (DEGs) and their annotation; Table S5: Information on selected DEGs for qPCR validation; Figure S1: Volcano plot (A) and hierarchical cluster analysis (B) of differentially expressed genes in response to 7-methylcoumarin; Figure S2: KEGG analysis of DEGs.
