# Supplementary material for: Insecticidal Activity and Mechanism of 7-Methylcoumarin Against Diaphorina citri Reveal the Role of Chemosensory Genes in Defense Response
Source: Insects. 2026 Oct 12;17(10):1045. doi: 10.3390/insects17101045 (PMC13705207; doi:10.3390/insects17101045)
Supplement: Supplementary file 1 [file insects-17-01045-s001.zip › Table S1 Primers used in this study.pdf]

Table S1 The information of all primers used in this study.

ID	Gene name	Primer sequence (5'–3')
Dcitr08g02940	Odorant binding protein 4	qF: CCCCTCAGCTGAAGGTCTAG
		qR: TTTGGTCGCTCACACAGTTG
Dcitr02g16710	Chemosensory protein 3	qF: CAGCTCAGAAGAAACAGGCC
		qR: CCACAGGCAGTCTTCAGGA
Dcitr13g06590	Chemosensory protein 7	qF: CCTTCTCTCCTACGTTGCCA
		qR: GCTAGAGCGTCTGGGATAGT
Dcitr08g07270	Larval cuticle protein A1A	qF: AGCCAGCTGAGGAGTATGAC
		qR: CTTTTGGATCGGCGGTGTAG
Dcitr03g21110	Cuticle protein	qF: TACGTCGCCCCTGTTTACAA
		qR: AATCCGTTGTGTTCGTCAGC
Dcitr01g05740	Cytochrome P450	qF: ACTTCAAAACATGCCCGTCC
		qR: AGGAAGCGGTCTGGATTGAA
Dcitr05g15850	ABC transporter, ATP-binding protein	qF: CAAGAATCACGGCTCAGCTC
		qR: TCTCCACGTATCGACAGCTC
Dcitr04g05950	Transforming growth factor-beta-induced protein ig-h3	qF: TCCAGGTCCTAACGGTGAAC
		qR: GGCTAAATCGTCCGTCCCTA
Dcitr04g11210	Succinate--CoA ligase [ADP-forming] subunit beta	qF: GGTCTGGTTAACGCTTGCAA
		qR: TTTACTGGCAGCCTCATCCA
Dcitr04g14290	Pro-resilin	qF: TCCCTACCCTCTACACACGA
		qR: ATGATCTCCTCCTCCCCACT
Dcitr06g09950	Protein yellow	qF: GCTCTGATCTTCCCCAGTGA
		qR: TACCTCGCACAGCTTCTTGA
Dcitr10g06650	Beta-glucuronidase	qF: ATCGTACGTCCCATTACCCC

		qR: TCGTTAGCCACAGACCACAT
Dcitr02g18920	Chemosensory protein 2	qF: ACATTGAGGCGGTTCTCAAC
		qR: TCCTTCTTGAGTCGCCTGAT
Dcitr00g05660	Hemicentin-2	qF: ACAGCAACTACTCGGACGAA
		qR: GTGATGGCCTCGACAATTCC
Dcitr03g04910	Phosphotransferase	qF: GTGCTCTCTGACCACCTCTT
		qR: CCATGCGGAACTTTTCACCA
Dcitr05g16620	Hamartin	qF: CTGAAGCTGTGGATGATGCC
		qR: CAACTGTACGGCATGTTCCC
Dcitr07g10110	BTB/POZ domain-containing protein	qF: GTAGTCCCGTCTTCATGGCT
		qR: AAGCAGAGTCATCGGGGAAA
Dcitr11g08060	General transcription factor 3C polypeptide 3	qF: AGCCCTGTCTGTTCTGAGTC
		qR: TCTCGTAGCTTTGTCCCGTT
Dcitr02g18920	Chemosensory protein 2	dsF: **TAATACGACTCACTATAGGG**CGTACCATCCTCCTATCACTGCT
		dsR: **TAATACGACTCACTATAGGG**CTTGTCTGTTTGACTTGGCGTG
Dcitr08g02940	Odorant binding protein 4	*SalI*-F: ACGCGTCGACAACAAACTCAACACAGGCCC
		NotI-R: ATAAGAATGCGGCCGCTTATGCTTGAACTTCACAGAACT

Bold letters indicate the T7 promoter sequence and the underline is denoted as the restriction site
