# Supplementary material for: Insecticidal Activity and Mechanism of 7-Methylcoumarin Against Diaphorina citri Reveal the Role of Chemosensory Genes in Defense Response
Source: Insects. 2026 Oct 12;17(10):1045. doi: 10.3390/insects17101045 (PMC13705207; doi:10.3390/insects17101045)
Supplement: Supplementary file 1 [file insects-17-01045-s001.zip › Table S2 Median lethal concentration (LC50) of 7-methylcoumarin against D. citri nymphs.pdf]

Table S2. The contact toxicity activity of 7-methylcoumarin against *D. citri* nymphs

**Compound**	**LC_{50} (mg/mL)**	**Confidence interval**	**Regression equation**	**χ^2**
7-methylcoumarin	0.40	0.29~0.60	y=1.52x + 0.61	21.53

LC_{50}, Median lethal concentration; χ^2, Chi-square value
