# Supplementary material for: Insecticidal Activity and Mechanism of 7-Methylcoumarin Against Diaphorina citri Reveal the Role of Chemosensory Genes in Defense Response
Source: Insects. 2026 Oct 12;17(10):1045. doi: 10.3390/insects17101045 (PMC13705207; doi:10.3390/insects17101045)
Supplement: Supplementary file 1 [file insects-17-01045-s001.zip › Table S3 Summary of transcriptome sequencing data.pdf]

Table S3 Quality control and data statistics of transcriptomes in *Diaphorina citri*.

Sample	Total Raw Reads	Total Clean Reads	Clean Reads Q20 (%)	Clean Reads Ratio (%)	GC Bases Ratio (%)
Ace1	38,955,690	38,633,722	99.26	99.17	36.74
Ace2	41,436,462	41,069,118	99.21	99.11	37.17
Ace3	45,689,936	45,329,744	99.29	99.21	36.36
Ace4	50,774,258	50,401,828	99.29	99.27	38.10
m7Cou1	55,241,236	54,717,724	99.18	99.05	37.72
m7Cou2	48,402,800	48,083,356	99.35	99.34	38.78
m7Cou3	41,603,330	41,233,028	99.21	99.11	36.55
m7Cou4	41,056,346	40,703,920	99.23	99.14	37.26
