# Supplementary material for: Insecticidal Activity and Mechanism of 7-Methylcoumarin Against Diaphorina citri Reveal the Role of Chemosensory Genes in Defense Response
Source: Insects. 2026 Oct 12;17(10):1045. doi: 10.3390/insects17101045 (PMC13705207; doi:10.3390/insects17101045)
Supplement: Supplementary file 1 [file insects-17-01045-s001.zip › Table S5 Information on selected DEGs for qPCR validation.pdf]

Table S5 The information of 10 DEGs used in qPCR validation.

Gene ID	Gene name	Ace	m7Cou	log_2FoldChange	p-Value
Dcitr08g02940	Odorant binding protein 4	1828.30	461.04	-1.99	0.014
Dcitr02g16710	Chemosensory protein 3	4664.66	2017.03	-1.21	0.00025
Dcitr13g06590	Chemosensory protein 7	24.47	9.77	-1.3	0.015
Dcitr08g07270	Larval cuticle protein A1A	8181.10	2267.59	-1.85	0.015
Dcitr03g21110	Cuticle protein	1339.18	485.71	-1.46	0.019
Dcitr02g18920	Chemosensory protein 2	1.37	9.63	2.81	0.014
Dcitr00g05660	Hemicentin-2	14.39	33.63	1.23	0.013
Dcitr03g04910	Phosphotransferase	8.09	20.63	1.35	0.018
Dcitr05g16620	Hamartin	9.42	23.71	1.33	0.013
Dcitr11g08060	General transcription factor 3C polypeptide 3	0.52	4.45	3. 10	0.025
